# Co-expression of HER2 and HER3 receptor tyrosine kinases enhances invasion of breast cells via stimulation of interleukin-8 autocrine secretion

**DOI:** 10.1186/bcr3329

**Published:** 2012-10-12

**Authors:** Nicola Aceto, Stephan Duss, Gwen MacDonald, Dominique S Meyer, Tim-C Roloff, Nancy E Hynes, Mohamed Bentires-Alj

**Affiliations:** 1 Mechanisms of Cancer Department, Friedrich Miescher Institute for Biomedical Research, Maulbeerstrasse 66, Basel, CH-4058 Switzerland; 2 Current address: Helen Diller Family Comprehensive Cancer Center, University of California at San Francisco, 1450 3rd Street, San Francisco, California 94158, USA

## Abstract

**Introduction:**

The tyrosine kinase receptors HER2 and HER3 play an important role in breast cancer. The HER2/HER3 heterodimer is a critical oncogenic unit associated with reduced relapse-free and decreased overall survival. While signaling cascades downstream of HER2 and HER3 have been studied extensively at the level of post-translational modification, little is known about the effects of HER2/HER3 overexpression and activation on gene expression in breast cancer. We have now defined the genetic landscape induced by activation of the HER2/HER3 unit in mammary cells, and have identified interleukin (IL)8 and CXCR1 as potential therapeutic targets for the treatment of HER2/HER3-overexpressing breast cancers.

**Methods:**

Three-dimensional (3D) cultures, invasion and migration assays were used to determine the effects of HER2 and HER3 co-expression and activation. Gene expression analysis was performed to identify the gene network induced by HER2/HER3 in 3D cultures. Bioinformatic analysis and neutralizing antibodies were used to identify key mediators of HER2/HER3-evoked invasion.

**Results:**

Co-expression of the tyrosine kinase receptors HER2 and HER3 induced migration and invasion of MCF10A cells. Microarray analysis of these cells revealed a specific "HER2/HER3 signature" comprising 80 upregulated transcripts, with IL8 being the highest (11-fold upregulation). Notably, examination of public datasets revealed high levels of IL8 transcripts in HER2-enriched as well as basal-like primary breast tumors, two subtypes characterized by a particularly poor prognosis. Moreover, IL8 expression correlated with high tumor grade and ER-negative status. Importantly, treatment with IL8-neutralizing antibodies prevented invasion of MCF10A-HER2/HER3 and BT474 cells in 3D cultures, highlighting the importance of IL8 autocrine signaling upon HER2/HER3 activation.

**Conclusions:**

Our findings demonstrate that HER2 and HER3 co-expression induces IL8 autocrine signaling, leading to the invasion of mammary cells. Agents targeting IL8 or its receptor CXCR1 may be useful for the treatment of HER2/HER3/IL8-positive breast cancers with invasive traits.

## Introduction

HER2 (ErbB2) and HER3 (ErbB3) are members of a family of four receptor tyrosine kinases that also includes the epidermal growth factor receptor (HER1/EGFR) and HER4 (ErbB4) [1]. HER2 overexpression accounts for approximately 20% of all breast cancers and is commonly associated with a poor prognosis [2]. The importance of HER2 in cancer is highlighted by the clinical efficacy of the anti-HER2 humanized monoclonal antibody trastuzumab (Herceptin), especially when combined with chemotherapy, for the treatment of HER2-overexpressing breast cancers [3,4]. HER3 has been strongly implicated as a dimerization partner of HER2 in promoting malignant transformation [5], mainly via activation of the PI3K pathway [6,7]. The HER2/HER3 heterodimer, stabilized by heregulin (HRG), has been identified as a strong oncogenic unit in breast cancer [5,8] and is associated with reduced relapse-free and decreased overall survival [9].

In HER2-dependent breast cancer cells, loss of HER3 reduces cell proliferation and decreases PI3K activity [10,11]. Moreover, inhibition of HER2 phosphorylation by tyrosine kinase inhibitors (TKIs) targeting EGFR and HER2 triggers feedback overexpression and activation of HER3 [12], thereby limiting the inhibitory effect of HER-targeting TKIs. These studies highlight a central role for HER3 in HER2-mediated breast tumorigenesis and response to therapy.

The signaling pathways activated by HER2 and HER3 have been studied extensively [13]. For example, while HER2 generally activates the Ras-MAPK, PLCγ-PKC, SHP2, STATs, PI3K/AKT and Src-dependent pathways [7], HER3 contains six binding sites for the p85 subunit of PI3K, which allow potent activation of the PI3K/AKT pathway [7]. In contrast, little is known about the effect of the HER2/HER3 oncogenic unit on gene expression in breast cancer.

In this study, using three-dimensional cultures, migration and invasion assays, genetic tools and bioinformatic analysis, we have defined the genetic landscape induced by simultaneous expression of HER2 and HER3 in mammary cells. These results reveal IL8 and its receptor CXCR1 as potential therapeutic targets for the treatment of HER2/HER3-overexpressing breast cancers.

## Materials and methods

### Monolayer and three-dimensional cultures

MCF10A cells were obtained from J Brugge (Harvard Medical School, Boston, MA, USA) and propagated in DMEM/F12 medium (Invitrogen, Carlsbad, CA, USA) supplemented with 5% horse serum (Hyclone, Logan, UT, USA), 20 ng/ml EGF (Peprotech, Rocky Hill, NJ, USA), 0.5 μg/ml hydrocortisone (Sigma, St. Louis, MO, USA), 100 ng/ml cholera toxin (Sigma, St. Louis, MO, USA), 10 μg/ml insulin (Sigma, St. Louis, MO, USA), 100 IU/ml penicillin and 100 μg/ml streptomycin. MCF10A cells were grown in three-dimensional cultures as described previously [14]. BT474 cells were obtained from the American Type Culture Collection (ATCC, Manassas, VA, USA) and grown in DMEM medium supplemented with 10% fetal bovine serum (Sigma, St. Louis, MO, USA). For three-dimensional cultures, BT474 cells were grown in DMEM medium containing 1% fetal bovine serum and 10 ng/ml heregulin (Sigma, St. Louis, MO, USA).

### Migration and invasion assays

Migration assays were performed using BD Chambers as per the manufacturer's instructions. Briefly, cells were starved overnight in DMEM/F12 containing 0.1% BSA and then seeded in the upper chamber of collagen-coated inserts at a concentration of 10,000 cells in 200 μl DMEM/F12 containing 0.1% BSA. The lower chamber was filled with 750 μl DMEM/F12 containing 0.1% BSA and 100 ng/ml heregulin. The cells were incubated for 24 h at 37°C in 5% CO2, at which point, cells remaining in the upper chamber were removed using a cotton swab. Cells on the lower surface of the membrane were fixed with 4% paraformaldehyde (PFA), stained with crystal violet and imaged. The same protocol was followed for the invasion assays, using BD BioCoat growth factor reduced Matrigel invasion chambers (BD Biosciences, San Jose, CA, USA), however the cells were not starved and were seeded in the upper chamber at 25,000 per well.

### Reagents

Heregulin (Sigma, St. Louis, MO, USA) 10 ng/ml and growth factor reduced Matrigel basement membrane matrix (BD Biosciences, San Jose, CA, USA) were used for three-dimensional cultures, and TRIzol reagent (Invitrogen, Carlsbad, CA, USA) for RNA extraction. The neutralizing antibodies anti-CXCR1, anti-CXCR2 and anti-IL8 (R&D Systems, Minneapolis, MN, USA) were used according to the manufacturer's instructions. Antibodies anti-Ki67 used for assessment of proliferation of MCF10A cells grown in two- and three-dimensional cultures were purchased from Thermo Scientific, Waltham, MA, USA. Human recombinant IL8 was purchased from Invitrogen, Carlsbad, CA, USA.

### Retroviral infection

MCF10A cells were infected overnight with retroviral particles containing pWZL-Hygro-hHER2 or pBabe-Neo-hHER3 at multiplicity of infection (MOI) = 10 in the presence of 8 μg/ml polybrene (Sigma, St. Louis, MO, USA), and selected with 250 μg/ml G418 (Gibco, Carlsbad, CA, USA) and/or 100 μg/ml hygromycin (Invivogen, San Diego, CA, USA).

### Microarray analysis

Total RNA was extracted from tumors with TRIzol reagent (Invitrogen, Carlsbad, CA, USA). Total RNA (300 ng) was processed with GeneChip WT cDNA Synthesis Kit and the GeneChip WT Terminal Labeling Kit (Affymetrix, Santa Clara, CA, USA), hybridized for 16 h to GeneChip Human Gene 1.0 ST arrays (Affymetrix, Santa Clara, CA, USA) and washed and scanned on a GeneChip Scanner 3000 with autoloader according to the manufacturer's instructions. Expression values were normalized and probeset-level values calculated with robust multi-array average (RMA) as implemented in the R/Bioconductor package, affy (R version 2.8). Contrasts between MCF10A-HER2/HER3 and the other groups were statistically analyzed in Genedata Expressionist (version 5.1). Venn diagrams were created for probesets with an absolute linear fold change > 1.5 and a *P*-value < 0.05 to find probesets commonly up- or downregulated in all contrasts. CEL files have been deposited in the GEO repository (GSE37009). Lists of differential genes including fold-changes and *P*-values were uploaded to Ingenuity Pathway analysis to study biological network enrichment.

### Analysis of IL8 expression in publicly available breast cancer datasets

We performed gene set analysis (GSA) of IL8 on primary breast tumors with the Gene Expression-based Outcome of Breast Cancer Online (GOBO) platform as described previously [15]. Examination of IL8 expression and its association with clinical parameters was conducted on a merged dataset comprising 1,881 primary breast tumor samples obtained from multiple experiments and classified into breast cancer subtypes by two different classifiers [16,17].

### Real-time PCR

Total RNA was extracted with TRIzol reagent and used as a template for production of cDNA. The cDNA was used in SYBR-based quantitative real-time PCR for quantification of IL8, E-cadherin, N-cadherin, vimentin and fibronectin1 transcript levels. IL8 forward primer: 5'-AAGCTGGCCGTGGCTCTCTT-3' and reverse primer: 5'-TGGTGGCGCAGTGTGGTCCA-3'. E-cadherin forward primer: 5'-TGCCCAGAAAATGAAAAAGG-3' and reverse primer: 5'-GTGTATGTGGCAATGCGTTC-3'. N-cadherin forward primer: 5'-ACAGTGGCCACCTACAAAGG-3' and reverse primer: 5'-CCGAGATGGGGTTGATAATG-3'. Vimentin forward primer: 5'-GAGAACTTTGCCGTTGAAGC-3' and reverse primer: 5'-GCTTCCTGTAGGTGGCAATC-3'. Fibronectin1 forward primer: 5'-CAGTGGGAGACCTCGAGAAG-3' and reverse primer: 5'-TCCCTCGGAACATCAGAAAC-3'. Glyceraldehyde-3-phosphate dehydrogenase (GAPDH) levels were used to normalize the data. The GAPDH forward primer was 5'-ACCCAGAAGACTGTGGATGG-3' and the GAPDH reverse primer 5'-TCTAGACGGCAGGTCAGGTC-3'.

## Results

### Co-expression of HER2 and HER3 induces mammary cell invasion, migration and proliferation in the presence of heregulin

**Figure 1:**
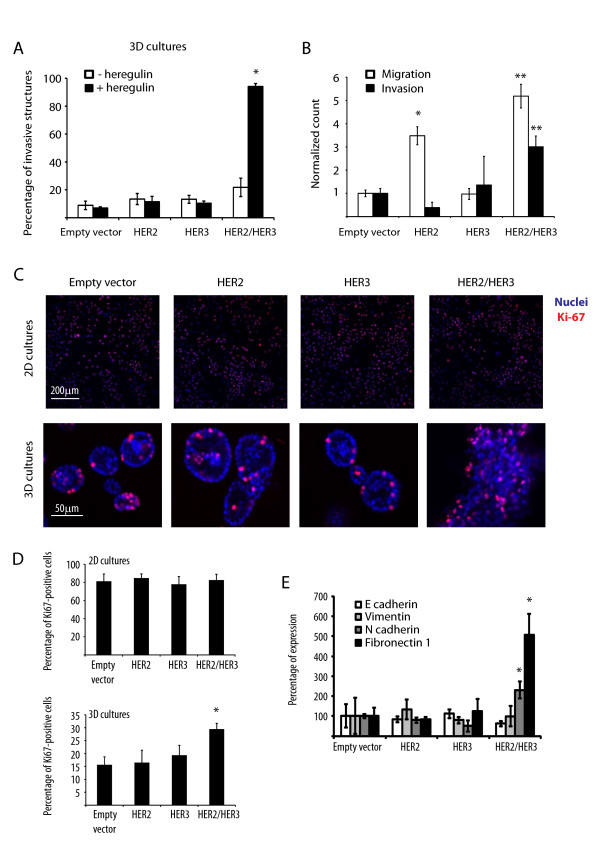
**HER2 and HER3 co-expression induces mammary cell invasion, migration and proliferation**. (**A**) Percentages of invasive structures in the presence or absence of heregulin 10 ng/ml. Results represent means ± standard error of the mean (SEM) (*n *= 3, *P < 0.05). (**B**) Normalized cell count for the migration and invasion assays. Results represent means ± SEM (*n *= 3, *P < 0.003, **P < 0.002). (**C**) Representative confocal images of equatorial cross-sections of MCF10A cells expressing empty vector, HER2, HER3 or HER2/HER3 grown in two- or three-dimensional cultures for 15 days in the presence of 10 ng/ml heregulin and stained with TO-PRO (blue) and anti-Ki67 antibodies (red). (**D**) Percentage of Ki67-positive cells when grown in two- or three-dimensional cultures. Results represent means ± SEM (*n *= 3, *P < 0.03). (**E**) Quantitative real-time PCR on RNA extracts of MCF10A cells expressing empty vector, HER2, HER3 or HER2/HER3 grown in two- or three-dimensional cultures for 15 days in the presence of 10 ng/ml heregulin. Quantitative real-time PCR was performed using primers against the epithelial-to-mesenchymal transition (EMT) markers E-cadherin, N-cadherin, vimentin and fibronectin1. Results represent means ± SEM (*n *= 3, *P < 0.05).

We expressed the receptor tyrosine kinases (RTKs) HER2 and HER3 into the immortalized but non-transformed mammary epithelial cells MCF10A. We then seeded MCF10A cells expressing empty vector, HER2, HER3, or HER2 plus HER3 (HER2/HER3) in three-dimensional cultures for 15 days in the presence or absence of heregulin, a known HER3-ligand that stabilizes and activates the HER2/HER3 heterodimer [5,6]. When grown in three-dimensional cultures, MCF10A cells expressing the empty vector formed hollow spheres with polarized cells, mimicking the morphology of normal mammary acinar structures (Figure S1 in Additional file 1). Interestingly, HER2 expression induced the formation of larger spheres that were polarized and characterized by a hollow lumen. Of note, HER3 expression alone had no major effect on MCF10A cells in three-dimensional cultures, with only a minor proportion of structures being multi-acinar with a filled lumen. In contrast, consistent with our previous observations [18], simultaneous expression of HER2/HER3 in MCF10A cells in the presence of heregulin induced the formation of highly invasive and unpolarized structures containing a filled lumen, while HER2/HER3 overexpression in the absence of heregulin did not (Figure 1A; Figure S1 in Additional file 1; data not shown). These data indicate that heregulin is required for HER2/HER3-induced invasion, loss of polarization and luminal filling in three-dimensional cultures of human mammary epithelial cells.

In addition, we performed a migration assay using the Boyden chamber as well as an invasion assay using Matrigel-coated chambers. In both cases, co-expression of HER2 and HER3 increased migration and invasion towards a heregulin-containing medium (Figure 1B and Figure S2 in Additional file 1). Of note, HER2 expression alone increased migration of MCF10A cells but had no effect on invasion (Figure 1B).

Moreover, we grew MCF10A cells expressing a control vector, HER2, HER3 or HER2/HER3 in two- or three-dimensional cultures for 15 days and assessed proliferation by staining with anti-Ki67 antibodies. While we found no difference in proliferation among cell lines when grown in two-dimensional cultures, we observed an increase in the percentage of Ki67-positive cells in MCF10A-HER2/HER3 grown in three-dimensional culture (Figure 1C and 1D). Moreover, we extracted RNA from MCF10A cells expressing an empty vector, HER2, HER3 or HER2/HER3 that were grown in three-dimensional cultures for 15 days and assessed expression levels of epithelial-to-mesenchymal transition (EMT) markers. While we found no change in expression of E-cadherin and vimentin, we observed significant upregulation of the mesenchymal markers N-cadherin and fibronectin1 (Figure 1E). Altogether, these results indicate that co-expression of HER2 and HER3 induces migration, invasion and proliferation of mammary cells in the presence of heregulin.

### Gene expression analysis of MCF10A-HER2/HER3 cells grown in three-dimensional cultures reveals the presence of a HER2/HER3-driven gene signature

The gene expression profile induced by HER2/HER3 co-expression in breast epithelial cells has not been examined previously. To this end, we extracted RNA from MCF10A cells expressing an empty vector, HER2, HER3, or HER2/HER3 grown in three-dimensional cultures for 15 days in the presence of heregulin and performed microarray analysis on the RNA obtained from the different groups to obtain gene expression contrasts of MCF10A-HER2/HER3 versus the other conditions. We identified a set of 157 Affymetrix probe set IDs differentially regulated in MCF10A-HER2/HER3 cells (fold change > 1.5-fold, *P *< 0.05) compared with all the other groups (Figure S3 in Additional file 1). Characterization of these IDs led to the identification of 137 differentially regulated genes, of which 80 were upregulated specifically in MCF10A-HER2/HER3 cells, and referred to as the HER2/HER3 signature (Figure S4 and Table S1 in Additional file 1). Gene ontology analysis of the HER2/HER3 signature with the Ingenuity^® ^Systems resource revealed enrichment in pathways involved in cellular movement, cell-to-cell signaling and interaction, cell death, cellular development and cellular growth and proliferation (Figure S5 in Additional file 1). These results indicate that the co-expression and activation of HER2/HER3 induces a specific gene signature associated with invasiveness. Among these genes, IL8 was upregulated approximately 11-fold and was the most upregulated gene in MCF10A-HER2/HER3 cells relative to all other conditions (Table S1 in Additional file 1). Furthermore, IL8 was found to be involved in all pathways identified by the Ingenuity resource (Figure S5 in Additional file 1; data not shown). Thus, IL8 may play an important role downstream of the HER2/HER3 heterodimer in breast cells.

### IL8 contributes to HER2/HER3-induced invasion in three-dimensional cultures

**Figure 2:**
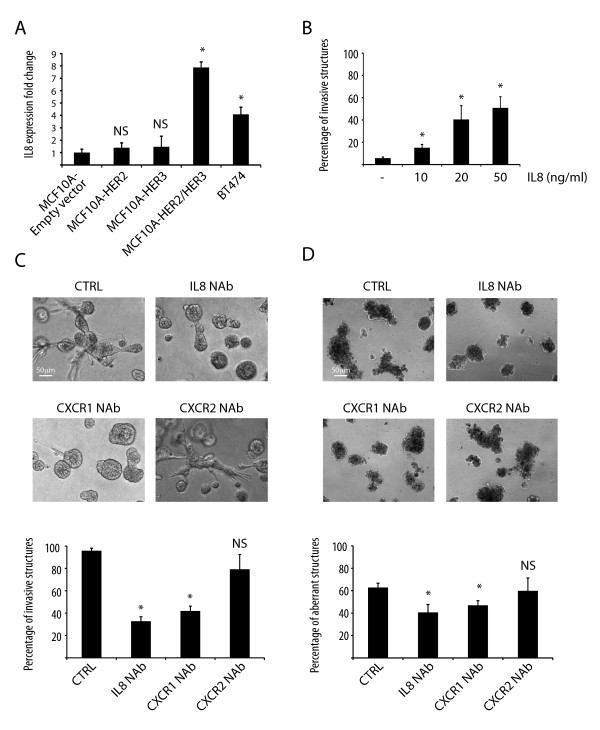
**IL8 or CXCR1 blockade reduces HER2/HER3-evoked invasion of mammary cells**. (**A**) Quantitative real-time PCR of IL8. The bar graph shows the percentages of IL8 expression in MCF10A cells expressing empty vector, HER2, HER3 or HER2/HER3, as well as in the HER2/HER3-positive breast cancer cell line BT474. Results represent means ± standard error of the mean (SEM) (*n *= 3, *P < 0.018; NS, not significant). (**B**) Percentage of invasive structures upon stimulation of MCF10A cells with IL8. Results represent means ± SEM (*n *= 3, *P < 0.05). (**C**) Representative phase-contrast pictures of MCF10A-HER2/HER3 cells grown in three-dimensional cultures for 15 days in the presence of heregulin and the indicated neutralizing antibodies. The bar graph shows the percentages of invasive structures. Results represent means ± SEM (*n *= 3, *P < 0.0004; NS, not significant). (**D**) Representative phase-contrast pictures of BT474 cells grown in three-dimensional cultures for 15 days in the presence of heregulin and neutralizing antibodies. The bar graph shows the percentages of aberrant structures. Results represent means ± SEM (*n *= 3; *P < 0.05; NS, not significant). CTRL, control.

Our results suggest that HER2/HER3-induced IL8 autocrine secretion contributes to the invasion of transformed breast cells. Given our finding that co-activation of HER2 and HER3 induces IL8 production, we compared IL8 expression levels in BT474 cells, a HER2- and HER3-positive breast cancer cell line, to MCF10A cells expressing an empty vector, HER2, HER3 or HER2/3. The IL8 transcript was highly expressed in MCF10A-HER2/HER3 as well as in BT474 cells (Figure 2A).

We then asked whether IL8 stimulation *per se *induces invasion of MCF10A cells grown in three-dimensional cultures. We seeded MCF10A cells in three-dimensional cultures and found that stimulation with increasing concentrations of IL8 induced invasion (Figure 2B and Figure S6 in Additional file 1), although not to the same extent as the co-expression of HER2 and HER3 in the presence of heregulin (Figure 1A). These results suggest that additional pathways downstream of HER2/HER3 are needed to achieve their higher invasion phenotype.

In addition, to test whether IL8 signaling contributes to HER2/HER3-evoked invasion, we grew MCF10A-HER2/3 and BT474 cells in three-dimensional cultures in the presence of an IL8-neutralizing antibody. Remarkably, blockade of IL8 autocrine signaling reduced the invasive behavior of these cells (Figure 2C, D). We also tested whether inhibition of the IL8 receptors CXCR1 and CXCR2 affected invasion. While there was no difference in invasion upon CXCR2 blockade, the MCF10A-HER2/3 and BT474 cells-invasive/aberrant phenotype was significantly reduced upon neutralization of CXCR1 (Figure 2C, D). These results indicate that IL8 autocrine signaling acts predominantly via CXCR1.

### IL8 is highly expressed in HER2-enriched and basal-like primary breast cancers

**Figure 3:**
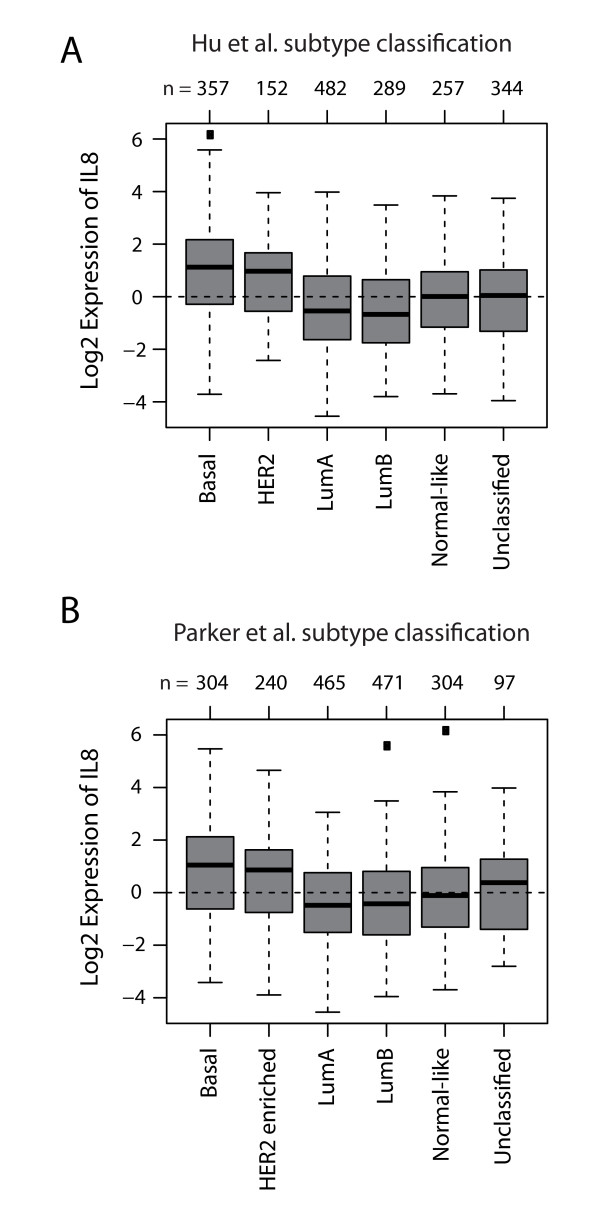
**IL8 expression correlates with HER2-enriched and basal breast cancers**. Gene set analysis (GSA) of IL8 expression in a dataset comprising 1,881 primary breast tumors http://co.bmc.lu.se/gobo. (**A**) Box plot of IL8 expression in breast tumor samples classified into six molecular subtypes according to the classifiers of Hu *et al. *[16]; *P *< 0.0001 by analysis of variance (ANOVA). (**B**) Box plot of IL8 expression in breast tumor samples classified into six molecular subtypes according to the classifiers of Parker *et al. *[17]; *P *< 0.0001 by ANOVA. Numbers above the charts report the number of patients in each subtype group.

To assess whether IL8 is overexpressed in human tumors, we analyzed IL8 expression levels in a public dataset containing gene expression data from, 1,881 primary breast tumors [15], classified by two independent classification methods [16,17]. Unequivocally, we found high expression of IL8 in HER2-enriched and basal breast cancers (Figure 3A, B), two subtypes associated with poor prognosis.

**Figure 4:**
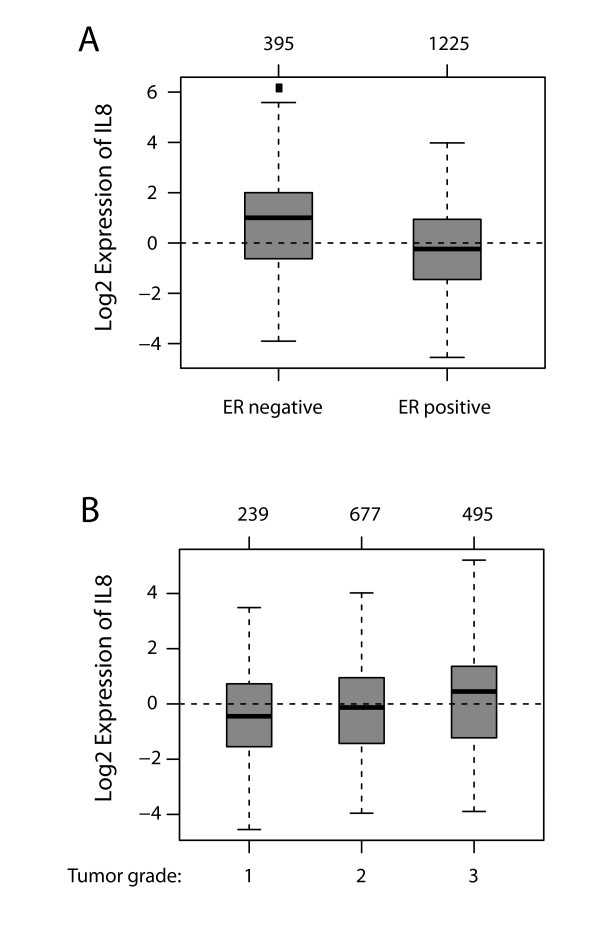
**IL8 expression correlates with ER-negative and high-grade primary breast tumors**. Gene set analysis (GSA) of IL8 in a dataset comprising 1,881 primary breast tumors http://co.bmc.lu.se/gobo. (**A**). IL8 expression correlates with estrogen receptor (ER)-negative tumors. Box plot of IL8 expression in ER-negative and ER-positive primary breast cancers; *P *< 0.0001 by analysis of variance (ANOVA). **B**. IL8 expression correlates with high tumor grade. Box plot of IL8 expression in grade 1, 2 and 3 primary breast cancers, respectively; *P *< 0.0001 by ANOVA. Numbers above the charts show the number of patients in each subtype group.

Next, we examined whether IL8 expression correlates with clinicopathological parameters such as estrogen receptor (ER) status and tumor grade in breast cancer patients. To this end, primary breast tumors were divided into ER-negative and ER-positive groups and IL8 expression was analyzed in each group. IL8 was significantly upregulated in ER-negative tumors (Figure 4A). In addition, primary breast tumors were divided into three groups according to tumor grade 1, 2, or 3, and IL8 expression was analyzed in each group. In line with our findings correlating IL8 with the aggressiveness of HER2/HER3 cells, we observed significant overexpression of IL8 in high-grade primary tumors (Figure 4B).

**Figure 5:**
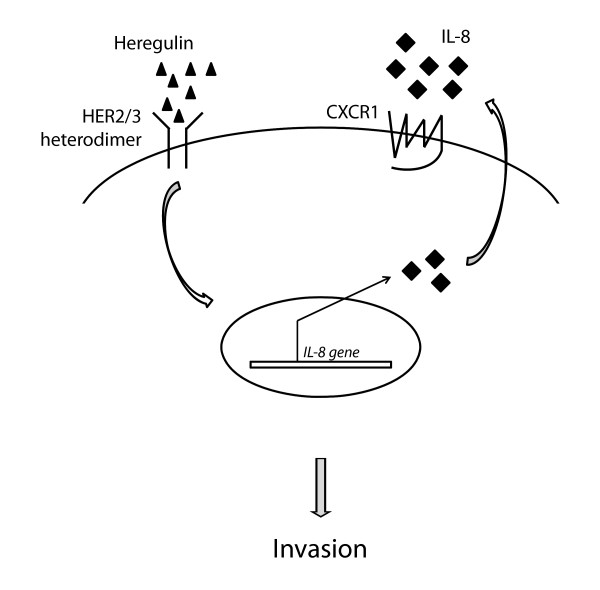
**Model of the HER2/HER3/IL8 signaling cascade**. Heregulin-induced heterodimerization and activation of HER2/HER3 causes upregulation of the IL8 transcript, which in turn mediates invasion via CXCR1.

In summary, our data show that activation of the HER2/HER3 oncogenic unit upregulates several genes including IL8, which in turn acts via CXCR1 to induce invasion of mammary epithelial cells (Figure 5). We also show that high IL8 expression correlates with ER-negative status and high tumor grade. These results provide new insights into the changes in gene expression upon activation of the HER2/HER3 oncogenic unit and point to IL8 and its receptor CXCR1 as possible therapeutic targets for the treatment of invasive HER2/HER3/IL8-positive breast cancers.

## Discussion

The signaling cascades activated by the tyrosine kinase receptors HER2 and HER3 have been studied extensively in the neoplastic breast [7]. In contrast, little is known about the effects of HER2/HER3 co-activation on gene expression. We have used three-dimensional cultures of mammary cells and bioinformatic analysis to specifically investigate this aspect. We found that activation of the HER2/HER3 oncogenic unit causes upregulation of several genes including the chemokine IL8, which was discovered originally as a chemotactic factor for leukocytes [19] and shown later to contribute to human cancer progression through its potential mitogenic, motogenic and angiogenic functions [20].

Following HER2/HER3 co-activation in mammary cells, we identified 80 upregulated genes. While in this study we found IL8 to be an important factor mediating invasion downstream of HER2/HER3, we showed previously that knockdown of another of these upregulated genes, the zinc finger E-box binding homeobox 1 (ZEB1), also blocks invasion in MCF10A-HER2/HER3 cells [18]. These results highlight the fact that several genes of the HER2/HER3 signature may contribute to the invasive phenotype induced by co-activation of HER2/HER3 receptors. Our study reveals novel potential targets for the treatment of HER2- and HER3-positive human breast tumors.

We show here that IL8 upregulation is a consequence of HER2/HER3 co-activation in human mammary cells. Elevated IL8 levels have previously been described in breast tumor specimens relative to normal breast tissue [21]. We analyzed public datasets and found high IL8 expression specifically in HER2-positive and basal-like tumors. It is conceivable that HER2/HER3-independent mechanisms may be responsible for high IL8 expression in some triple negative breast cancers (TNBCs) [20].

Interestingly, other studies have demonstrated that the IL8/CXCR1 signaling is important for breast cancer cell invasion [22] and predominantly active in cells with the cancer stem cell (CSC) phenotype when compared to non-CSCs [23,24]. Moreover, blockade of CXCR1 significantly reduces the CSC population, leading to decreased tumorigenicity and metastasis [23,24]. In line with these findings, we have previously reported that co-expression of HER2 and HER3 in MCF10A cells increases the proportion of cells with the CSC phenotype [18]. Altogether, these results highlight an important role for HER2/HER3/IL8 in the regulation of invasion and the CSC phenotype.

We observed a correlation between high IL8 expression levels and negative ER status as well as high tumor grade. In addition to breast cancer [21], IL8 is overexpressed in other types of cancers, including colon [25], gastric [26], melanoma [27,28], ovarian [29,30], pancreatic [31] and prostate cancer [32]. Generally, IL8 expression has been shown to contribute to human cancer progression [20,33]; however, the mechanisms regulating IL8 expression have remained obscure. For example, stimuli such as lipopolysaccharide, phorbol 12-myristate 13-acetate, IL1 and TNF can induce IL8 production [20], and stress factors such as hypoxia, acidosis, nitric oxide and cell density have also been shown to influence IL8 expression in particular cell types [20]. Here, we demonstrate a direct relationship between HER2/HER3 activation and IL8 expression in breast cancer. It is conceivable that the activity of the HER2/HER3 heterodimer also promotes IL8 expression in cancers of different origins.

## Conclusions

Our study provides evidence for a link between HER2/HER3 overexpression and IL8 production in mammary cells. First, we show that co-activation of HER2 and HER3 causes upregulation of IL8, which in turn contributes to HER2/HER3-evoked cellular invasion. Moreover, we show that the IL8 transcript is highly expressed in HER2-enriched and basal-like human primary breast cancers, and that high IL8 levels correlate with ER-negative status and high tumor grade. Finally, treatment with IL8- or CXCR1-neutralizing antibodies reduced invasive behavior of HER2/HER3-driven malignant breast cells, highlighting a role for IL8 downstream of activated HER2/HER3. Our results identify IL8 and its receptor CXCR1 as potential targets for the treatment of breast cancers with active HER2/HER3/IL8 signaling.

## Abbreviations

ANOVA: analysis of variance; BSA: bovine serum albumin; CSC: cancer stem cell; DMEM: Dulbecco's modified Eagle's medium; EGFR: epidermal growth factor receptor (HER1); EMT: epithelial-to-mesenchymal transition; ER: estrogen receptor; GAPDH: glyceraldehyde-3-phosphate dehydrogenase; GOBO: Gene Expression-based Outcome of Breast Cancer Online; GSA: gene set analysis; HRG: heregulin; MOI: multiplicity of infection; PCR: polymerase chain reaction; PFA: paraformaldehyde; RMA: robust multi-array average; RTK: receptor tyrosine kinase; SEM: standard error of the mean; TKI: tyrosine kinase inhibitors; TNBC: triple negative breast cancers; TNF: tumor necrosis factor.

## Competing interests

Some of the information in this publication is related to a patent application by The Friedrich Miescher Institute for Biomedical Research. MB-A and NA are listed as inventors on this application. All other authors declare no competing interests.

## Authors' contributions

NA carried out three-dimensional cultures, RNA extraction, real-time PCR and blocking antibody experiments. DSM generated retroviral constructs. SD and TCR performed computational biology experiments and generated the microarray data. GM performed migration and invasion assays. NEH discussed the data and the manuscript. NA and MBA wrote the main body of the manuscript. All authors have read and approved the manuscript for publication.

## Supplementary Material

Additional file 1 **HER2 and HER3 co-expression induces invasion, migration and a specific gene signature**. Figure S1. Representative phase-contrast images of MCF10A cells expressing empty vector, HER2, HER3 or HER2 plus HER3 (HER2/HER3) and grown in three-dimensional cultures for 15 days in the presence of heregulin. Figure S2. Representative phase-contrast images from migration and invasion assays with MCF10A cells expressing empty vector, HER2, HER3 or HER2/HER3. Figure S3. Venn diagram showing the number of Affymetrix IDs obtained when comparing gene expression data of MCF10A-HER2/HER3 cells to the other conditions (empty vector, HER2 or HER3). A total of 157 Affymetrix IDs are differentially regulated in MCF10A-HER2/HER3 cells. Figure S4. The 157 Affymetrix IDs associated with MCF10A-HER2/HER3 cells correspond to 137 genes, of which 80 are upregulated (upon HER2/HER3 co-activation) and are referred to as the HER2/3 signature (see also Table S1). Figure S5. Bar graph obtained with gene ontology analysis (Ingenuity) showing the major pathways of the HER2/3 signature and associated log P values. The number of HER2/3 signature genes involved in each pathway is indicated in round brackets. Figure S6. Representative phase-contrast pictures of MCF10A cells grown in three-dimensional cultures for 15 days in the presence or absence of IL8. Table S1. Fold change (upregulation) of each of the HER2/HER3 signature genes listed in alphabetical order (*P *< 0.05). Click here for file
