# Supplementary material for: Co-expression of HER2 and HER3 receptor tyrosine kinases enhances invasion of breast cells via stimulation of interleukin-8 autocrine secretion
Source: Breast Cancer Res. 2012 Oct 12;14(5):R131. doi: 10.1186/bcr3329 (PMC4053109; doi:10.1186/bcr3329)
Supplement: Additional file 1 — HER2 and HER3 co-expression induces invasion, migration and a specific gene signature. Figure S1. Representative phase-contrast images of MCF10A cells expressing empty vector, HER2, HER3 or HER2 plus HER3 (HER2/HER3) and grown in three-dimensional cultures for 15 days in the presence of heregulin. Figure S2. Representative phase-contrast images from migration and invasion assays with MCF10A cells expressing empty vector, HER2, HER3 or HER2/HER3. Figure S3. Venn diagram showing the number of Affymetrix IDs obtained when comparing gene expression data of MCF10A-HER2/HER3 cells to the other conditions (empty vector, HER2 or HER3). A total of 157 Affymetrix IDs are differentially regulated in MCF10A-HER2/HER3 cells. Figure S4. The 157 Affymetrix IDs associated with MCF10A-HER2/HER3 cells correspond to 137 genes, of which 80 are upregulated (upon HER2/HER3 co-activation) and are referred to as the HER2/3 signature (see also Table S1). Figure S5. Bar graph obtained with gene ontology analysis (Ingenuity) showing the major pathways of the HER2/3 signature and associated log P values. The number of HER2/3 signature genes involved in each pathway is indicated in round brackets. Figure S6. Representative phase-contrast pictures of MCF10A cells grown in three-dimensional cultures for 15 days in the presence or absence of IL8. Table S1. Fold change (upregulation) of each of the HER2/HER3 signature genes listed in alphabetical order (P < 0.05). [file bcr3329-S1.PDF]

S1

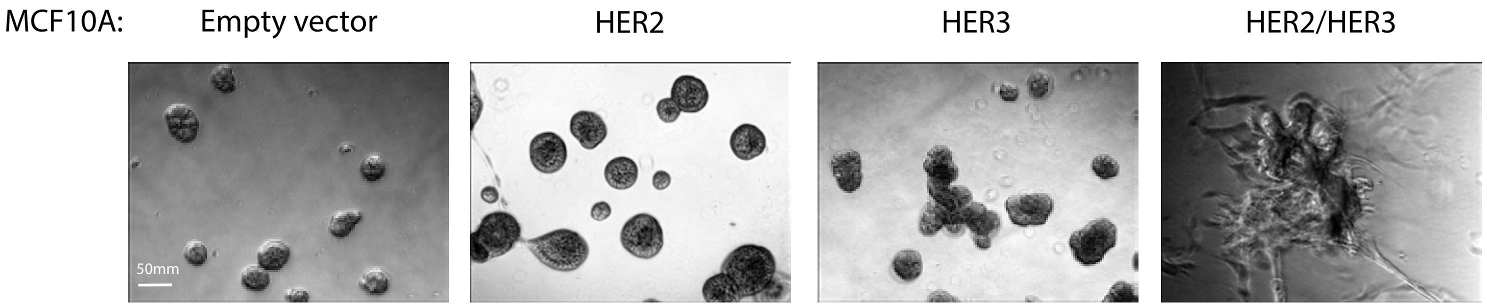
MCF10A:
Empty vector
HER2
HER3
HER2/HER3
50mm

S2

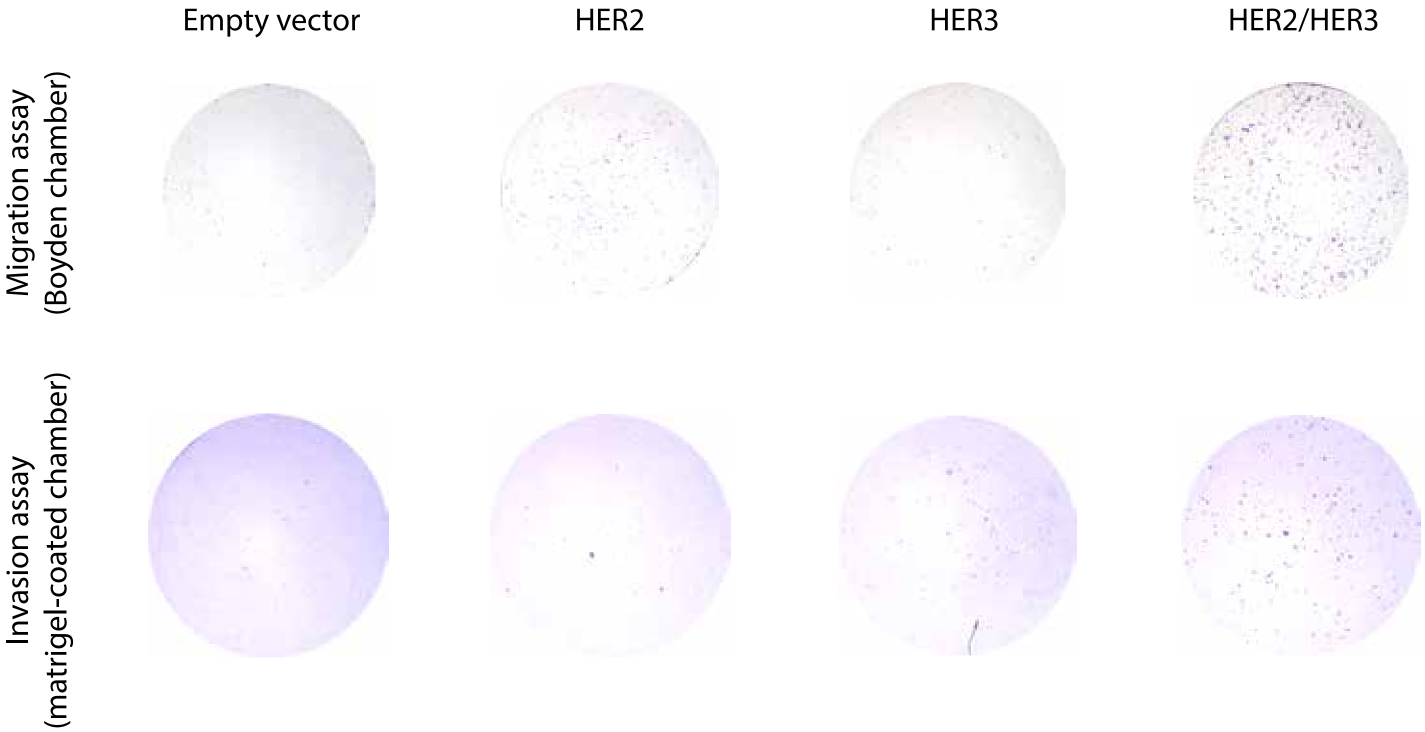
Empty vector
HER2
HER3
HER2/HER3
Migration assay
(Boyden chamber)
Invasion assay
(matrigel-coated chamber)

S3

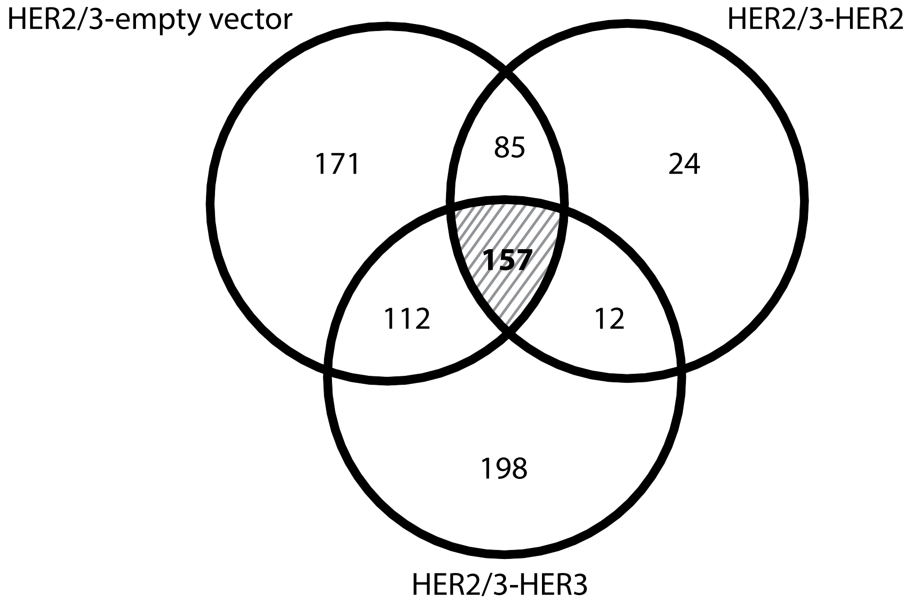
HER2/3-empty vector
HER2/3-HER2
171
85
24
157
112
12
198
HER2/3-HER3

S4

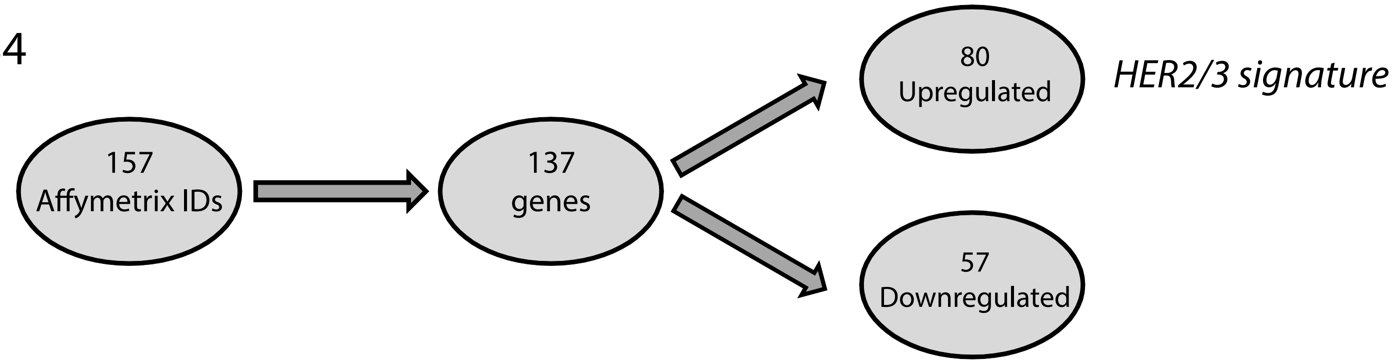
157
Affymetrix IDs
137
genes
80
Upregulated
57
Downregulated
HER2/3 signature

S5

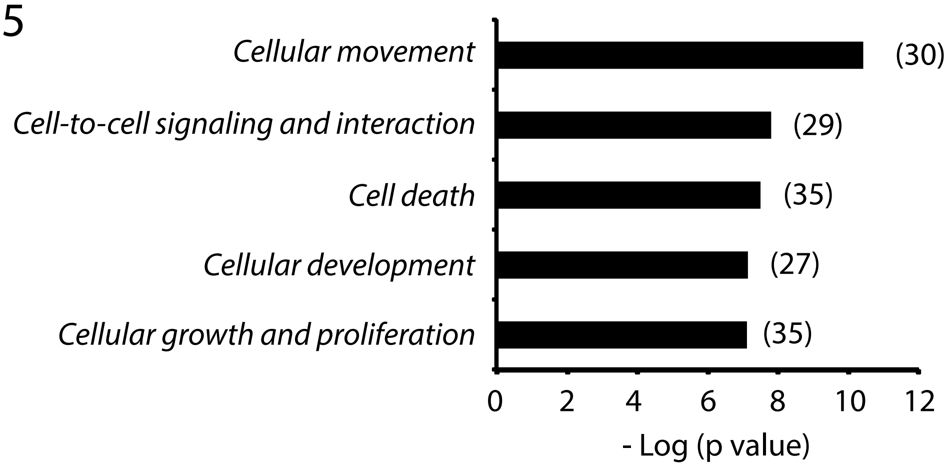
Cellular movement
Cell-to-cell signaling and interaction
Cell death
Cellular development
Cellular growth and proliferation
(30)
(29)
(35)
(27)
(35)
0
2
4
6
8
10
12
- Log (p value)

S6

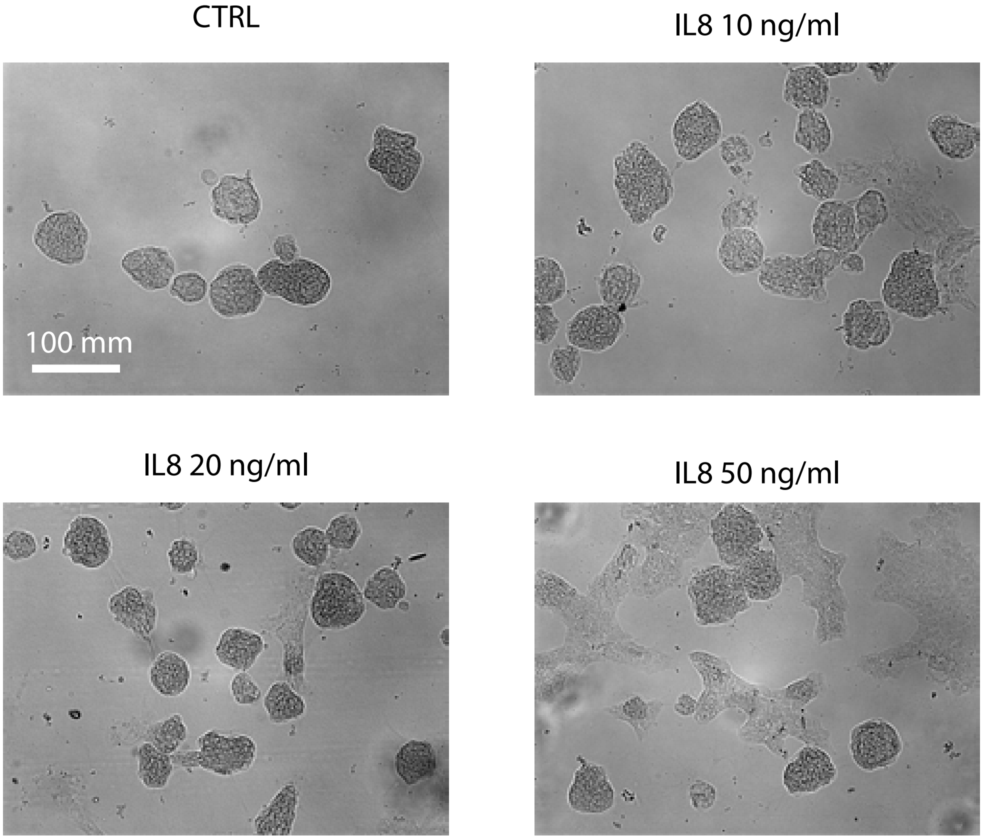

Table S1. Fold change (upregulation) of each of the HER2/HER3 signature genes listed in alphabetical order ($P< 0.05$).

Fold change	Symbol	Entrez gene name
3.158	ABCD2	ATP-binding cassette, sub-family D (ALD), member 2
2.012	ABLIM3	actin binding LIM protein family, member 3
2.018	ACSL4	acyl-CoA synthetase long-chain family member 4
2.899	ALDH1L2	aldehyde dehydrogenase 1 family, member L2
4.677	AOX1	aldehyde oxidase 1
3.975	AREG	amphiregulin
2.468	ASNS	asparagine synthetase (glutamine-hydrolyzing)
1.770	BCL2L1	BCL2-like 1
5.318	C12ORF39	chromosome 12 open reading frame 39
2.753	C17ORF60	chromosome 17 open reading frame 60
2.758	C6ORF138	chromosome 6 open reading frame 138
1.949	CCL20	chemokine (C-C motif) ligand 20
2.662	CDH2	cadherin 2, type 1, N-cadherin (neuronal)
2.021	CMTM7	CKLF-like MARVEL transmembrane domain containing 7
3.093	CNTN3	contactin 3 (plasmacytoma associated)
2.240	CPD	carboxypeptidase D
2.857	CSF3	colony stimulating factor 3 (granulocyte)
1.788	CTSB	cathepsin B
6.312	CXCL1	chemokine (C-X-C motif) ligand 1 (melanoma growth stimulating activity, alpha)
2.105	CYP4X1	cytochrome P450, family 4, subfamily X, polypeptide 1
2.794	DAB2	disabled homolog 2, mitogen-responsive phosphoprotein (Drosophila)

6.582	ERBB2	v-erb-b2 erythroblastic leukemia viral oncogene homolog 2, neuro/glioblastoma derived oncogene homolog (avian)
2.630	ERBB3	v-erb-b2 erythroblastic leukemia viral oncogene homolog 3 (avian)
2.312	ETV1	ets variant 1
3.547	ETV5	ets variant 5
2.265	F3	coagulation factor III (thromboplastin, tissue factor)
2.781	FADS1	fatty acid desaturase 1
2.733	FAP	fibroblast activation protein, alpha
6.679	FN1	fibronectin 1
4.047	FPR1	formyl peptide receptor 1
1.920	GARS	glycyl-tRNA synthetase
1.909	GPD2	glycerol-3-phosphate dehydrogenase 2 (mitochondrial)
1.607	HIPK2	homeodomain interacting protein kinase 2
2.132	HMGA2	high mobility group AT-hook 2
1.689	IARS	isoleucyl-tRNA synthetase
2.011	IFI6	interferon, alpha-inducible protein 6
11.659	IL8	interleukin 8
1.702	INHBA	inhibin, beta A
3.757	ITGA10	integrin, alpha 10
2.153	ITGA5	integrin, alpha 5 (fibronectin receptor, alpha polypeptide)
2.361	KDELC2	KDEL (Lys-Asp-Glu-Leu) containing 2
1.795	KDELR3	KDEL (Lys-Asp-Glu-Leu) endoplasmic reticulum protein retention receptor 3
2.526	KYNU	kynureninase (L-kynurenine hydrolase)
3.211	LAMB3	laminin, beta 3
2.487	LTBP1	latent transforming growth factor beta binding protein 1

1.773	MAN2A1	mannosidase, alpha, class 2A, member 1
3.124	MCTP1	multiple C2 domains, transmembrane 1
1.721	MDM2	Mdm2 p53 binding protein homolog (mouse)
1.835	MTHFD2	methylenetetrahydrofolate dehydrogenase (NADP+ dependent) 2, methenyltetrahydrofolate cyclohydrolase
1.986	MTHFD2L	methylenetetrahydrofolate dehydrogenase (NADP+ dependent) 2-like
2.580	NRCAM	neuronal cell adhesion molecule
1.955	PDCD1LG2	programmed cell death 1 ligand 2
3.505	PHGDH	phosphoglycerate dehydrogenase
3.223	PITPNC1	phosphatidylinositol transfer protein, cytoplasmic 1
2.049	PTPRG	protein tyrosine phosphatase, receptor type, G
5.504	RERG	RAS-like, estrogen-regulated, growth inhibitor
1.916	RPGR (includes EG:6103)	retinitis pigmentosa GTPase regulator
1.647	RPGRIP1L	RPGRIP1-like
3.042	RPS6KA2	ribosomal protein S6 kinase, 90 kDa, polypeptide 2
2.677	SERPINA1	serpin peptidase inhibitor, clade A (alpha-1 antiproteinase, antitrypsin), member 1
3.913	SERPINE2	serpin peptidase inhibitor, clade E (nexin, plasminogen activator inhibitor type 1), member 2
2.815	SLC16A4	solute carrier family 16, member 4 (monocarboxylic acid transporter 5)
1.922	SLC1A1	solute carrier family 1 (neuronal/epithelial high affinity glutamate transporter, system Xag), member 1
3.743	SLC26A2	solute carrier family 26 (sulfate transporter), member 2
3.501	SLC39A8	solute carrier family 39 (zinc transporter), member 8
1.954	SMTN	smoothelin

1.843	SNORD116-6	small nucleolar RNA, C/D box 116-6
3.095	SPRY4	sprouty homolog 4 (Drosophila)
6.713	SRGN	serglycin
3.109	SRPX	sushi-repeat-containing protein, X-linked
2.784	STC2	stanniocalcin 2
1.593	TARS	threonyl-tRNA synthetase
2.012	TFPI	tissue factor pathway inhibitor (lipoprotein-associated coagulation inhibitor)
2.009	TIMP1	TIMP metallopeptidase inhibitor 1
2.346	TLR4	toll-like receptor 4
1.648	TMEM55A	transmembrane protein 55A
3.047	TSPAN5	tetraspanin 5
2.335	VNN1	vanin 1
2.912	XDH	xanthine dehydrogenase
1.885	ZEB1	zinc finger E-box binding homeobox 1
